# Influence of Electronic Modulation of Phenanthroline-Derived Ligands on Separation of Lanthanides and Actinides

**DOI:** 10.3390/molecules27061786

**Published:** 2022-03-09

**Authors:** Ming-Ming Li, Xiao-Juan Liu, Qi Yang, Yue-Kun Liu, Jiang Nie, Shu-Ming Yang, Ya-Ya Yang, Fu-Yan Lou, Song-Tao Xiao, Ying-Gen Ouyang, Guo-An Ye

**Affiliations:** Department of Radiochemistry, China Institute of Atomic Energy, P.O. Box 275, Beijing 102413, China; sfsvoyager@163.com (M.-M.L.); liuxj0106@163.com (X.-J.L.); yangqi_96@163.com (Q.Y.); alston980780@foxmail.com (Y.-K.L.); niejiang_ciae@163.com (J.N.); shumingyang_ciae@163.com (S.-M.Y.); yangyaya_ciae@163.com (Y.-Y.Y.); loufuyan1989@163.com (F.-Y.L.); xiao_songtao@126.com (S.-T.X.); ouyang_yinggen@163.com (Y.-G.O.)

**Keywords:** separation, solvent extraction, lanthanides, ligand, phenanthroline

## Abstract

The solvent extraction, complexing ability, and basicity of tetradentate N-donor 2,9-bis(5,5,8,8-tetramethyl-5,6,7,8-tetrahydro-1,2,4-benzotriazin-3-yl)-1,10-phenanthroline (CyMe_4_-BT- Phen) and its derivatives functionalized by Br, hydroxyphenyl, nitryl were discussed and compared. It was demonstrated that four BTPhen ligands are able to selectively extract Am(lll) over Eu(lll). It was notable that the distribution ratio of 5-nitryl-CyMe_4_-BTPhen for Eu(lll) was suppressed under 0.02, which was much lower compared to D_Eu(lll)_ = 1 by CyMe_4_-BTPhen. The analysis of the effect of the substituent on the affinity to lanthanides was conducted by UV/vis and fluorescence spectroscopic titration. The stability constants of various ligands with Eu(lll) were obtained by fitting titration curve. Additionally, the basicity of various ligands was determined to be 3.1 ± 0.1, 2.3 ± 0.2, 0.9 ± 0.2, 0.5 ± 0.1 by NMR in the media of CD_3_OD with the addition of DClO_4_. The basicity of ligands follows the order of L_1_ > L_2_ > L_3_ > L_4_, indicating the tendency of protonation decreases with the electron-withdrawing ability increase.

## 1. Introduction

In the reprocessing of spent fuel, minor actinides such as Americium, Neptunium, and Curium dominate the long-term radiotoxicity of high-level liquid waste (HLLW) after the PUREX reprocessing of uranium and plutonium. It is essential to shorten the lifetime of these radionuclides and minimize the volumes of waste for storage and isolation away from biosphere. One strategy currently being pursued is Partitioning and Transmutation (P&T) [1,2,3,4,5], whereby the radioactive nuclides are first separated from non-radioactive lanthanides by the Selective ActiNides EXtraction (SANEX) process and then converted into short-lived or stable elements by neutron-induced fission. This separation is indispensable since lanthanides with higher neutron capture cross sections will absorb neutrons in preference to actinides, resulting in an inefficient utilization of neutrons [6,7]. The separation of radioactive actinides from lanthanides is therefore a primary challenge in the process of nuclear fuel reprocessing for efficient transmutation. However, the similarities in chemical properties such as ionic radius and coordination models between two groups of f-block elements make the separation hard to achieve [8,9]. It is acknowledged that there is a greater availability of valence orbitals in the actinides leading to more covalent bonding natures in the actinide–ligand than those of lanthanides [10,11].

**Figure 1:**
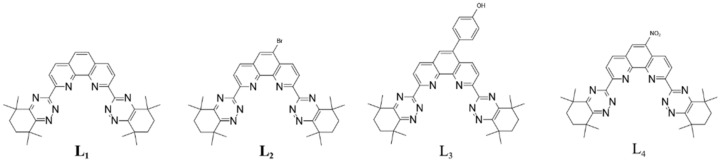
Structures of four Bis-Triazine Phenanthrolines.

In recent years, many ligands have been developed and investigated for their extraction performance, coordination models, resistance to hydrolysis, and radiolysis [12,13,14,15]. It was found that ligands containing N-donor atoms or S-donor atoms could discriminate trivalent actinides from trivalent lanthanides. Due to second waste generated by α, β, and γ radiolysis during the use of ligands containing S-donor atoms, the development of N-donor ligands has been the priority. Among these N-donor ligands, 2,6-bis(5,6-dialkyl-1,2,4-triazin-3-yl) pyridines (BTPs) [16,17,18] and 6,6′-bis(5,6-dialkyl-1,2,4-triazin-3-yl)-2,2′-bipyridines (BTBPs) [19,20,21,22], have been extensively studied and considered the promising N-donor ligands for the continuous separation of actinides and lanthanides since they are able to directly extract actinides from nitric acid solution. It has been demonstrated in SANEX that BTBP is suitable for the separation of actinides and lanthanides at laboratory scale. Unfortunately, the kinetics of extraction using CyMe_4_-BTBP was found to be rather slow necessitating the addition of a phase-transfer agents such as TODGA (*N*,*N*,*N*’,*N*’-tetra-octyl-diglycolamide) or DMDOHEMA (*N*,*N*’-dimethyl-*N*,*N*’-dioctyl-2-hexylethoxymalonamide) to improve the extraction kinetics [20,23]. This disadvantage led to the development of CyMe_4_-BTPhen (L_1_ as depicted in Figure 1) [24]. In this molecule, the bipyridine moiety is replaced by a phenanthroline moiety thus prescribing the cis-conformation that is less favored in the BTBP ligand but essential for metal ion coordination. The reason for the improved kinetics may be attributed to a large dipole moment in the phenanthroline framework, making it able to coordinate with water via hydrogen bonds at the interface during its extraction [24]. The conformational rigidity and juxtaposition of N-donor atoms endows L_1_ optimum extraction thermodynamics and kinetics. Unfortunately, the affinity of L_1_ for Am is so high that it leads to difficult stripping of Am(lll) (HNO_3_ = 0.1 mol/L, D_Am_ = 17, (Ligand) = 10 mmol/L in n-octanol). Based on that, ligands with Br and 4-hydroxyphenyl substitution at the 5 position (5-bromine-2,9-bis(5,5,8,8-tetramethyl-5,6,7,8-tetrahydro-1,2,4-benzotriazin-3-yl)-1,10-phenanthroline L_2_ (Figure 1) and 5-(4-hydroxyphenyl)-2,9-bis(5,5,8,8-tetramethyl-5,6,7,8-tetrahydro-1,2,4-benzotriazin-3-yl)-1,10-phenanthroline L_3_ (Figure 1) were synthesized and investigated for their extraction ability of actinides and lanthanides in this paper [25]. In order to gain further insight to elucidate reasons for the differences between these BTPhen ligands including a novel substituted structure L_4_ (Figure 1), the comparison of solvent extraction under same conditions, complexing ability, and protonation inclination were carried out in our work.

## 2. Results and Discussion

### 2.1. Solvent Extraction Study

**Figure 2:**
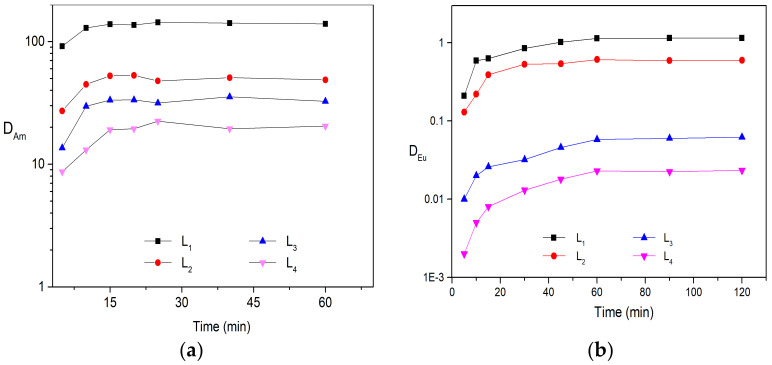
Extraction results of Am and Eu: (**a**) Description of dependence of Am(lll) distribution ratios on balance time. Organic phase: 5 mmol/L BTPhen/n-octanol; Aqueous phase: trace amount of ^241^Am in 1 mol/L HNO_3_ solution, Contact time, 5–60 min; (**b**) Description of dependence of Eu(lll) distribution ratios on balance time. Organic phase: 5 mmol/L BTPhen/n-octanol; Aqueous phase: 0.1 mmol/L Eu (NO_3_)_3_ in 1 mol/L HNO_3_ solution, Contact time, 5–120 min (Temperature, 298 ± 0.1 K).

Liquid–liquid solvent extraction was carried out to determine the distribution ratio of Am (lll) and Eu(lll) by four substituted BTPhen ligands in the media of HNO_3_. The organic phases were prepared by dissolving four ligands into n-octanol and were contacted with nitric acid solution containing ^241^Am and inactive Eu, respectively. Various solvents (such as kerosene, 3-Nitrobenzotrifluoride, n-octanol, nitrobenzene, and dichloromethane) were tested for ligands; it turned out that n-octanol was the best selection for BTPhen ligands though L_4_ exhibited poor solubility in it. Based on this conclusion, 5 mmol/L extractant solution was employed for all ligands in order to compare the extraction performance under the same concentration. The dependence of distribution ratio on extraction balance time for Am (lll) and Eu(lll) was confirmed in advance to obtain the equilibrium time. The results indicate that the types of substitution have no influence on the dynamic extraction of Bis-Triazine Phenanthroline toward Am(lll) and Eu(lll). BTPhen ligand L_1_–L_4_ almost simultaneously reach extraction equilibrium for Eu(lll) after 60 min vibration and 15 min for Am(lll) as shown from Figure 2. 

According to the extraction behaviors of four Bis-Triazine Phenanthroline ligands toward Am (lll) and Eu (lll), the distribution ratio value of Am by ligand L_1_ and L_2_ is considerably high even only 5 mmol/L ligand was employed in the organic phase. In addition, the affinity of ligands to Am is significantly stronger than Eu, SF_Am/Eu_ for ligands L_1_, L_2_, L_3_, and L_4_ are 130, 83, 550, and 870, respectively, which is roughly in agreement with the results in the previous reports [24,25]. Due to excellent separation performance, extremely fast extraction rates, and strong extraction ability of ligand L_1_ for minor actinide (Am as representation) and lanthanides, it has been considered the promising candidate to treat HLLW in the SANEX process. However, the distribution ratio for Am even at low acidic conditions (D_Am_ = 17, HNO_3_ = 0.1mol/L, (ligand) = 0.01 mol/L) is still very high leading to difficult stripping from extracted organic phase for subsequent step. 

It has been reported that the extraction ability of Bis-Triazine Phenanthroline mainly originated from two phenanthroline-N atom coordinating with most lanthanide ions [26]. The average of metal–ligand bond length acquired from crystal structures such as Eu-N_phen_ = 2.515 is shorter than Eu-N_triazinyl_ = 2.541, implying that there is a greater interaction between lanthanides ions and phenanthroline N-donor atoms than triazinyl N-donor atoms [26].

Quantum chemical calculation of complexes of various BTPhen ligands withAm/Eu also showed the similar results [27]. Thus, it might be effective to weaken the electronic density of phenanthroline skeleton via inductive effect exerted by substituted group Br, hydroxyphenyl, nitryl. It is observed that the order of affinity to metal ion from extraction results inversely follows the electron-withdrawing ability of substitution on phenanthroline: L_1_ > L_2_ > L_3_ > L_4_.

**Figure 3:**
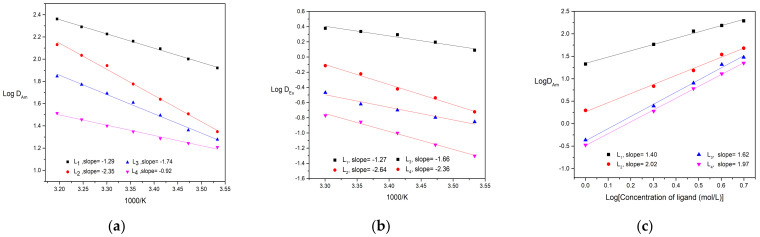
Extraction results of Am and Eu–dependence of Am(lll) and Eu(lll) distribution ratios on extraction temperature and ligand concentration: (**a**) Description of dependence of Am(lll) on extraction temperature. Organic phase: 5 mmol/L BTPhen/n–octanol; Aqueous phase: trace amount of ^241^Am in 1 mol/L HNO_3_ solution (Temperature, 298 ± 0.1 K; Contact time, 20 min); (**b**) Description of dependence of Eu(lll) distribution ratios on extraction temperature. Organic phase: 5 mmol/L BTPhen/n–octanol; Aqueous phase: 0.1 mmol/L Eu(NO_3_)_3_ in 1 mol/L HNO_3_ solution. (Temperature, 298 ± 0.1 K; Contact time, 60 min); (**c**): Description of influence of ligand concentration on the extraction of Am(lll). Organic phase: 1–5 mmol/L BTPhen/n–octanol; Aqueous phase: trace amount of ^241^Am in 1 mol/L HNO_3_ solution (Temperature, 298 ± 0.1 K; Contact time, 20 min).

The dependence of distribution ratio of Am(lll) and Eu(lll) for ligands on aqueous nitric concentration was similar to CyMe_4_-BTPhen reported in the previous work [24]. As the initial concentration of HNO_3_ in the aqueous phase increases, the distribution ratio toward metal ion goes up at first and then slightly declines at 2 mol/L HNO_3_. The increase of distribution ratio at first might be accounted for the growing number of ${\mathrm{NO}}_{3}^{-}$ present, which is in favor of formation of complexes. With continuing increase in nitric acid, the proportion of protonated ligands increases, resulting in available ligands decreases. The influence of substituent on the protonation inclination of BTPhen ligands will be investigated in the following section. The extraction performance of Bis-Triazine Phenanthroline ligands for Am(lll) and Eu(lll) as a function of extraction temperature are presented in Figure 3. It turns out that the extraction process of Am(lll) and Eu(lll) by four ligands belong to endothermic reaction. To probe the extraction mechanism, the influence of ligand concentration on the distribution ratio of Am(lll) was carried out and depicted in Figure 3. The plots of Log D_Am_ versus Log (L) of four types of BTPhen were obtained and gave four straight lines. Slopes analysis was employed to determine the number of ligands involved in the extracted complexes. The slopes of Ligand L_1_ and ligand L_3_ were around 2, indicating the type of 1:2 metal/ligand complexes during extraction by ligand L_1_ and L_3_ was formed. The crystal structure of [Eu(L_1_)NO_3_]^2+^ was obtained also verified our results [24]. Nevertheless, slopes of ligand L_2_ and L_4_, which differ from ligand L_1_ and L_3_, were between 1 and 2. Since crystal structure of maximum metal:ligand (1:2) of ligand 1 was formed, metal:ligand (2:3 or others) of homogeneous L_2_ and L_4_ was impossible due to the steric effect of many ligands. We considered that the formation of both 1:1 and 1:2 metal/ligand complexes was extracted into the organic phase during the extraction by ligand L_2_ and L_4_.

### 2.2. pKa Determination by NMR Titration

Since solvent extraction of reprocessing is accomplished in the media of HNO_3_ solution (2~3 mol/L), the basicity of ligands that determines the extent to which H^+^ ions compete with metal ions for ligands, is a very important property. Typically, the N-heterocyclics are readily prone to be protonated in highly acidic conditions. For example, Terpy(2,2′:6′,2′’-terpyridine) possesses the ability to extract Am from 0.1 mol/L HNO_3_ but fails to accomplish the extraction at 2 mol/L HNO_3_ [28,29]. This could be ascribed to highly nitric acid conditions caused by protonated Terpy, which is predominant, in which protonated ligands are transferred into the aqueous phase where their concentration in the organic phase is negligible in comparison to that of disprotonated species. However, the structures of easily protonated bilateral triazine and phenanthroline rings enable BTPhen ligands to extract metal ions from HNO_3_ solution. This phenomenon is mainly because the basicity of BTPhen ligands was strongly decreased by the conjugation effect between triazine and phenanthroline. So, it is meaningful to further investigate the effect of substitution on the basicity of BTPhen ligands.

**Figure 4:**
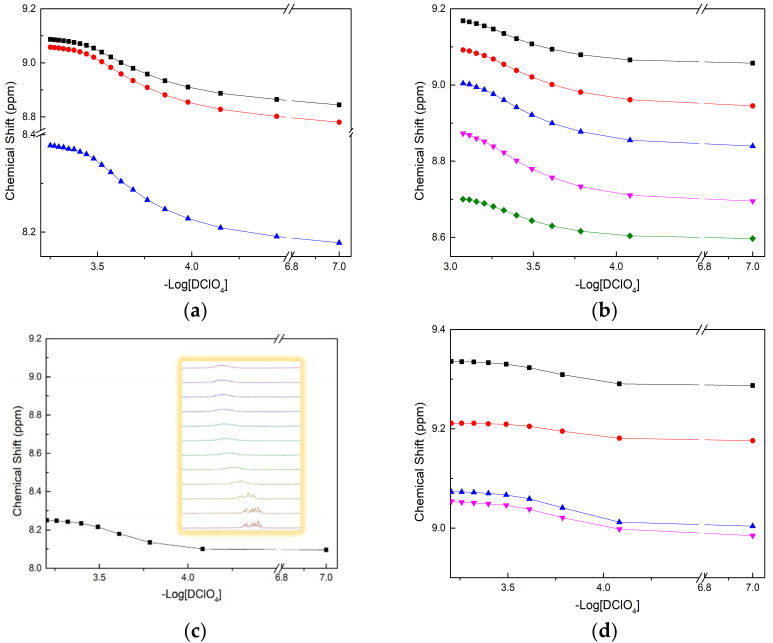
Plot of chemical shift (δ, ppm) as a function of pD for the H^1^ signals with addition of DClO_4_ in CD_3_OD: (**a**) Description of chemical shift in L_1_ (■—H at C3(or 8), ●—H at C4(or 7), ▲—H at C5 (or 6)); (**b**) Description of chemical shift in L_2_ (■—H at C8, ●—H at C3, ▲—H at C7, ▼—H at C4, ◆—H at C6); (**c**) Description of chemical shift in L_3_ (■—H at C6); (**d**) Description of chemical shift in L_4_ (■—H at C8, ●—H at C3, ▲—H at C7, ▼—H at C4).

The protonation constants of four BTPhen ligands were determined by recording the chemical shift in H^1^ with the titration of DClO_4_ [30,31]. The titration was not suspended until the chemical shift in H^1^ remain unchanged. Different amounts of acid were added into each solution to simulate one step in a titration, in which each solution represented a point in a typical titration. The calculated protonation constants of four BTPhen ligands are presented in Table 1. As we can see from Table 1, the original large dipole moment of phenanthroline makes it able to coordinate with H^+^ even with the effect of withdrawing electrons by biliteral triazine [32]. The protonation process caused the alteration of the chemical environment of H3, H4, H5, H6, H7, and H8 at the corresponding carbon C3, C4, C5, C6, C7, and C8. The chemical shift results of H5 or H6 on phenanthroline versus -Log [DClO_4_] are presented in Figure 4. Due to the symmetric property of L_1_, there are only three types of peak signal whose chemical shifts were visible in the spectra. As for L_2_, L_3_, and L_4_, chemical environment change caused by different substituted groups result in five types of peak signal appeared in the map. The reason for only one plot of chemical shifts presented in Figure 4c was that the shift signal of hydrogen on phenanthroline developed into unimodal from multimodal, which it is unable to identify. The peculiar phenomenon of peak signal of H in L_4_, which was weak at first, and then vanished, makes it hard to distinguish.

### 2.3. UV–vis Titration

**Figure 5:**
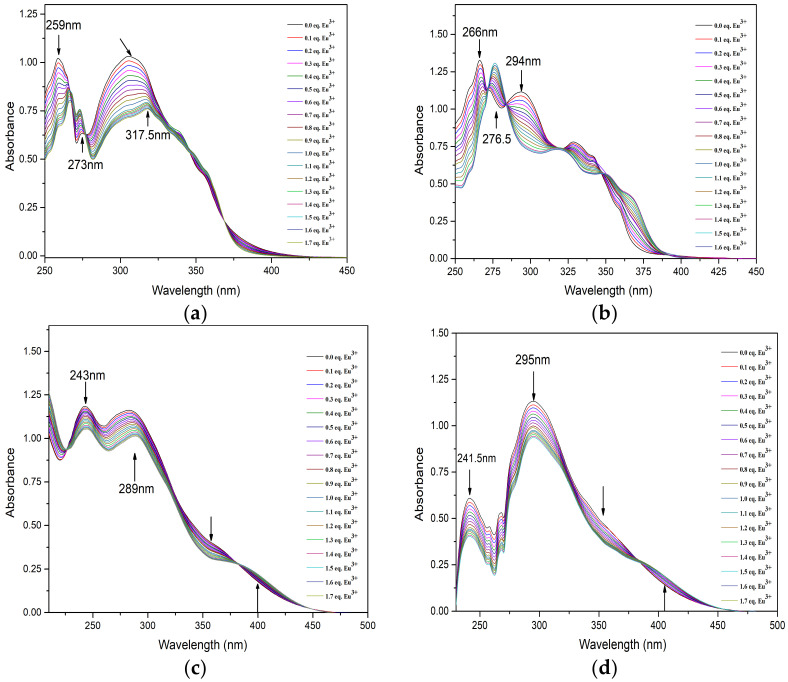
UV–visible absorption spectroscopic titrations of ligands ((**a**) for L_1_, (**b**) for L_2_, (**c**) for L_3_, (**d**) for L_4_) complexing with Eu^3+^ in CH_3_OH solution (T = 298 ± 0.1 K, I = 0.01 M Et_4_NNO_3_, initial condition (L_1_~L_4_) = 2 × 10^−5^ mol/L, V_ini_ = 2.00 mL, titrant condition [Eu(NO_3_)_3_] = 2 × 10^−4^ mol/L).

The complexation behavior of BTPhen ligands with lanthanides in methanol was investigated by UV–vis titration. The alteration of absorbance intensity of solution as a function of Eu(lll) concentration is shown in Figure 5. The absorption spectra of four ligands during the Ultra Visible region of 200–300 nm are dominated by the charge transfer of π–π* transition from the aromatic nature of the BTPhen structure. As Eu(lll) concentration increased in the L_1_ solution, the absorbance of ligands at 259 nm sharply decreased. The new peak at 265 nm and 273 nm emerged with the addition of up to 0.5 equivalent of Eu^3+^ corresponding to the M–L complex as presented in Figure 5a.

The increase in the absorption intensity for the new peak with the addition of Eu(lll) was also observed. The absorption at 265 nm shifted to 266 with the addition of Eu(lll) solution. It also could be seen that the peak at 317.5 nm apparently begun to form after one equivalent Eu^3+^ solution was added in the ligand solution. This indicates the 1:2 Eu^3+^/L_1_ complexes were formed. As for L_2_, sharp peak of spectrum at 266 nm and wide peak at 294 nm was obviously decreased as shown from Figure 5b. The absorption intensity at 276.5 nm was gradually enhanced and red-shifted upon the addition of Eu^3+^. This alteration on the behavior of spectra was analogous to the titration of L_1_. Similar results have been observed in the titration of L_3_ and L_4_, but more persuasive changes happened in the region from 325 to 425 nm. Moreover, two or more isosbestic points on every titration curve were generated, which confirms that more than one type of complex was formed upon the addition of Eu(lll). The stability constants of the 2:1 species of BTPhen ligands with Eu(lll) were determined to be 14.2 ± 0.5, 11.5 ± 0.3, 8.3 ± 0. 6, and 7.1 ± 0.4, respectively. It was found that the stability constants of Logβ_12_ the 2:1 complex formed between BTPhen ligands and trivalent europium ions increased from L_4_, L_3_, L_2_, to L_1_. This trend in the complexation strength agrees well the extraction properties of BTPhen for Eu as displayed in Figure 2a. It is reasonable to conclude that withdrawing substitution functionalized on the phenanthroline framework significantly decreased the complex ability of BTPhen ligands for trivalent lanthanides.

### 2.4. Fluorescence Spectroscopy

**Figure 6:**
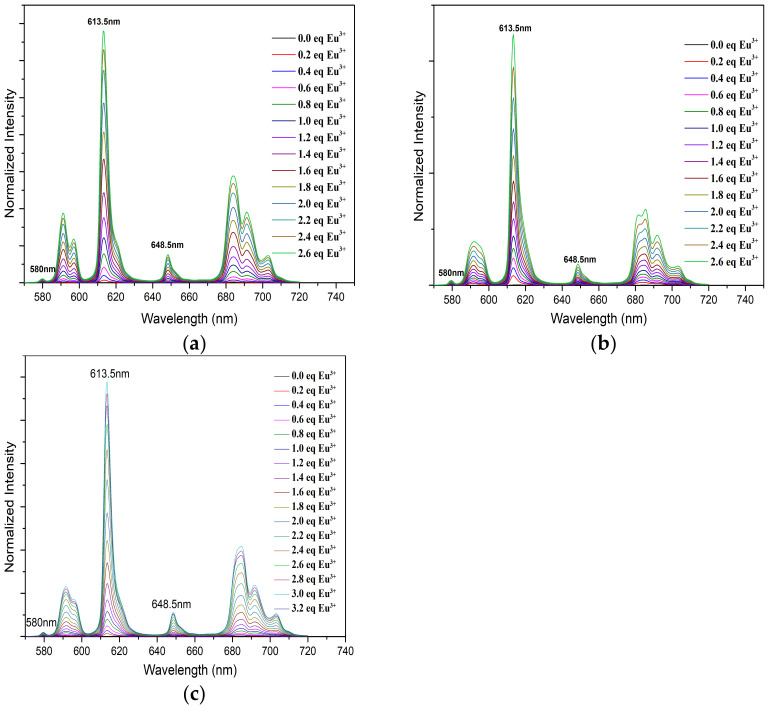
Normalized fluorescence emission spectra of Eu(III) in methanol with increasing BTPhen concentration. (Eu(lll))ini = 1 × 10^−5^ mol/L; V_ini_ = 2 mL; (L_1_, L_2_, L_4_) = 1 × 10^−3^ mol/L; 4 μL of ligand solution was added at one time. (**a**) Description of emission spectra of Eu (lll) with L_1_; (**b**) Description of emission spectra of Eu(lll) with L_2_; (**c**) Description of emission spectra of Eu(lll) with L_4_.

The fluorescence spectra of Eu(III) with CyMe_4_-BTPhen (a), 5-Br-CyMe_4_-BTPhen (b), and 5-(nitryl)-CyMe_4_-BTPhen (c) were illustrated in Figure 6. It is necessary to mention that there is no intensity increase in full spectra with the addition 5-(4-hydroxyphenyl)- CyMe_4_-BTPhen solution. The rigid skeleton and conjugation effect of phenanthroline should have enhanced the fluorescence intensity of Eu(lll) solution as observed in the titration of L_1_, L_2_, and L_4_. Nevertheless, the intensity of the spectra was nearly stationary while its complexation ability with Eu(lll) was stronger than L_4_ according to the investigation by UV/Vis. The reason for this “paradoxical phenomenon” in 5-(4-hydroxyphenyl)-CyMe_4_-BTPhen is still not fully understood and remains unsolved. The titration by Ligand L_1_, L_2_, and L_4_ exhibited remarkable enhancement in intensity of emissions across the addition of the corresponding ligand solution. A strongest intensity increase compared to the original Eu(lll) solution at 613.5 nm was observed resulting from ^5^D_0_→^7^F_2_ transitions [35]. The change in the remaining emission peaks in typical fluorescence spectra Eu (lll), which is at 580 nm, 594 nm, 648.5 nm, and 702 nm corresponding to ^5^D_0_→^7^F_0_, ^5^D_0_→^7^F_1_, ^5^D_0_→^7^F_3_, and ^5^D_0_→^7^F_4_ is relatively implicit [36,37]. The intensity plateau was reached for L_1_ and L_2_ after 2.6 μL equivalent ligands was added. As for L_4_, the spectra intensity did not stop growing until 2.4 μL of equivalent ligands was added. The stability constants of Eu(lll) with ligand 1, ligand 2, and ligand 4 by fitting spectra at 613.5 nm were determined to be 12.6 ± 0.4, 12.1 ± 0.8, and 8.6 ± 0.2, respectively. The calculated value of L_1_ with Eu(lll) was consistent with the previous report as shown from Table 2 [26]. The decrease in complexation ability of BTPhen ligands (except L_3_) with Eu(lll) indicates the affinity of ligands to lanthanides as the electron-withdrawing ability grows. This tendency was the manifestation of extraction results of BTPhen ligands toward Eu(lll).

## 3. Materials and Methods

### 3.1. Syntheses

**Figure 7:**
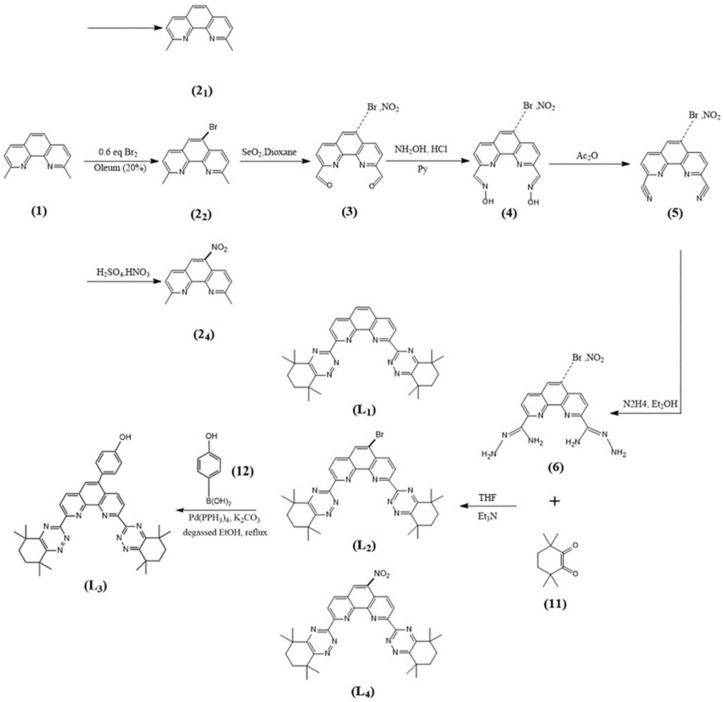
Syntheses of the BTPhen Ligand L1, L2, L3, and L4.

**Figure 8:**
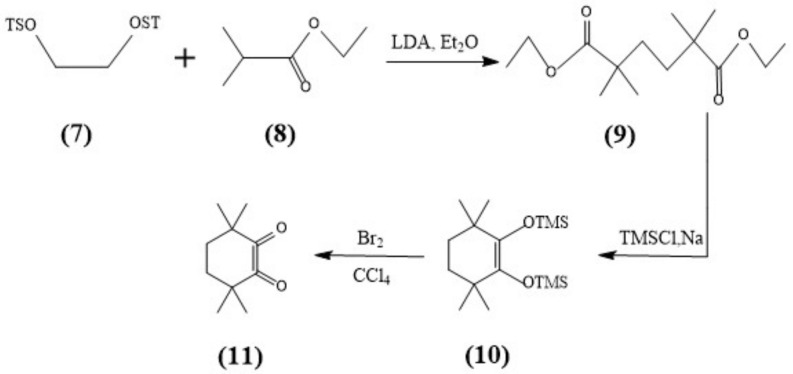
Synthesis of the Diketone (11).

The syntheses of CyMe_4_-BTPhen, 5-Br-CyMe_4_-BTPhen, and 5-(4-hydroxyphenyl)-CyMe_4_-BTPhen were achieved according to the route described by Lewis [24] and Afsar [25]. The difference in synthesis between 5-nitryl-CyMe_4_-BTPhen and CyMe_4_-BTPhen is 2,9-dimethyl-1,10-phenanthroline needs to be nitrified in the media of sulfuric acid preceding the synthesis of dialdehyde. The schemes of synthesis route of ligands were depicted in Figure 7 and Figure 8.

CyMe_4_-BTPhen: ^1^H NMR (400 MHz, CDCl_3_, ppm) δ 8.92 (d, J = 8.3 Hz, 2H), 8.61 (d, J = 8.4 Hz, 2H), 8.03 (s, 2H), 2.85 (s, 8H), 1.90 (s, 8H), 1.56 (d, J = 12.7 Hz, 24H). FT-IR (KBr, ν/cm-): 2864.48, 2932.80, 2960.50, 1363.05, 1385.681, 1151.89, 1249.23, 1640.78, 706.25, 1456.41, 1589.47, 1511.26, 840.33, 859.64, 869.89 MS(m/z):559.3298(M+H^+^), 581.3123(M+ Na^+^).

5-Br-CyMe_4_-BTPhen:^1^H NMR (400 MHz, CDCl_3_, ppm) δ 8.95 (d, J = 8.6 Hz, 1H), 8.89 (dd, J = 11.3, 8.4 Hz, 2H), 8.40 (d, J = 8.4 Hz, 1H), 8.29 (s, 1H), 2.62–2.26 (m, 8H), 1.90 (d, J = 2.7 Hz, 8H), 1.55 (dd, J = 11.9, 3.0 Hz, 24H).FT-IR (KBr, ν/cm-1): 2864.57, 2928.94, 2961.82, 1388.91, 1363.07, 1245.99, 1143.34, 705.44, 1641.98, 1514.61, 1602.92, 1602.9, 904.66, 866.63, 830.61, 632.65 MS(m/z):637.2409(M+H^+^),659.2231(M+Na^+^).

5-nitryl-CyMe_4_-BTPhen. ^1^H NMR (400 MHz, CDCl_3_, ppm) δ 9.24 (d, J = 8.8 Hz, 1H), 8.98 (dd, J = 21.1, 8.6 Hz, 2H), 8.81 (s, 1H), 8.64 (d, J = 8.3 Hz, 1H), 2.48 (s, 7H), 1.91 (s, 8H), 1.60–1.53 (m, 24H). FT-IR (KBr, ν/cm-1): 2863.24, 2929.24, 2960.67, 1363.72, 1384.77, 1123.05, 1247.46, 1508.88, 1630.01, 704.28cm-1, 1489.12, 1589.07, 843.80, 832.72, 771.54 MS(m/z): 604.3143 (M+H^+^), 626.2973 (M+ Na^+^).

5-(4-hydroxyphenyl)-CyMe4-BTPhen. 1H NMR (400 MHz, CDCl3, ppm): δ 8.89-8.90 (s,1H), δ 8.78-8,79(s,1H), δ 8.42-8,44 (s,1H), δ 8.30-8,31(s,1H), δ 7.68(s,1H), δ 6.64-6.66(d,2H), δ 6.48-6.49 (d,2H), δ 1.95 (m,8H), δ1.60-1.63(m,24H). FT-IR (KBr, ν/cm-1): 2864.42, 2928.68, 2960.25, 1368.76, 1388.07, 1144.54, 1244.18, 1612.78, 709.24, 1520.01, 1455.07, 1552.62, 839.13, 859.64, 869.89. MS(m/z): 651.3559(M+H+), 673.3379(M+ Na+).

### 3.2. Solvent Extraction

All the reagents supplied by Aladdin were used as received without further purification unless otherwise stated. Equal volumes (1 mL) of organic phase and aqueous phase were stirred for the extraction of Am(lll) and Eu(lll). The extraction of Am from HNO_3_ solution was performed at 298 ± 0.1 K. Nitric acid solution spiked with ^241^Am tracer amount was mixed with 0.5 mmol/L BTPhen ligands dissolved in n-octanol by Vortex oscillator for desired time (max. 120 min and min. 5 min). Temperature controlled extraction was carried out in the water bath magnetic stirrer with magneton in the plastic vials to maintain constant temperature during extraction. As for extraction of Eu(lll), it was conducted in the nonradioactive condition. The mixture for both Am and Eu extraction was centrifuged for 5 min to separate phases. The activity of ^241^Am and amount of Eu were separately determined by Tricarb 2910 tr liquid scintillator (Perkin Elmer company) and inductively coupled plasma atomic emission spectrometer ICP-AES. Distribution ratio of Am is determined by the ratio between the radioactivity counts of organic phase and aqueous phase after extraction. Distribution ratio of Eu(lll) is defined by the concentration of the Eu^3+^ in the organic phase divided by that in the aqueous phase. The separation factor (SF_Am/Eu_) was calculated by the ratio of distribution ratios between Am(lll) and Eu(lll).

### 3.3. pKa Determination

The protonation behavior of Bis-Triazine Phenanthrolines in the acid was investigated by NMR titration. Chemical shift was recorded by adding 0.1 mol/L DClO_4_ into 2 mmol/L ligand/ CD_3_OD solution. In the NMR measurements (ADVANCEⅢ, Bruker BioSpin GmbH), the magnetic field was stabilized by locking it to the D signal of the solvent. The sample temperatures were regulated to be 295 ± 0.1 K during all acquisitions. The titration was completed with addition of 90 μL L_1_, 65 μL L_2_, 60 μL L_3_, and 55 μL L_4_ with 5 μL at each time. The binding constants were determined using the hypNMR2008 software [39].

### 3.4. UV–vis Spectrometric Titration

The UV–vis spectroscopic titrations were conducted at 298 ± 0.1 K in the medium of methanol by Lambda950, Perkin Elmer. The stock solution was prepared by dissolving proper amounts of BTPhen ligands into methanol at concentration of 1.0 × 10^−3^ mol/L. Then ligand solution was diluted into 1.0 × 10^−5^ mol/L for use, and 2.0 mL BTPhen solution of 1.0 × 10^−5^ mol/L was added into quarts cell of 1 cm path length, then the initial spectrum was recorded. The each titration was performed by adding 1.0 × 10^−4^ mol/L Eu(NO_3_)_3_·6 H_2_O in methanol into ligand solution. Before the measurement, the mixture solution was vigorously shaken for 1 min to assure the complexation equilibrium. Stability constants were calculated by HypSpec program in the spectra of wavelength from 200 to 400 nm [40,41].

### 3.5. Fluorescence Spectroscopy

The fluorescence spectra of four BTPhen ligands with Eu (lll) were investigated in the cuvette cell of 1 cm path length at 298 ± 0.1 K by HORIBA Scientific. Ligand solution was prepared by dissolving 5.58 mg, 6.36 mg, 6.50 mg, and 6.03 mg into 10 mL methanol resulting in initial concentrations of 1.0 × 10^−3^ mol/L, respectively. Eu(NO_3_)_3_ stock solution was diluted into 1.0 × 10^−5^ mol/L for use. It has been reported that the complexation of CyMe_4_-BTPhen with f block elements exhibit slow kinetics, so the batch experiments were carried out with equilibrium of 32 h in Extraction Oscillator (200 rpm) at 298 ± 1 K [38]. The spectrum was recorded in the range of 570–720 nm with an interval of 0.2 nm with an excitation wavelength at 394 nm. The bandwidth of excitation and emission was set to be 5 nm and 2 nm. The stability constants of the complexes were determined by a nonlinear least-squares regression method using the HyperSpec program [40,41]. It is worthy to mention that emission spectra are recorded perpendicular to the laser beam with a time delay of 1 µs after excitation. This delay allows discrimination of the short-lived fluorescence of the organic ligand without compromising the analysis of the Eu(lll) complexes since the fluorescence lifetime of these species is considerably longer.

## 4. Conclusions

The four tetradentate N-donor ligands CyMe_4_-BTPhen, 5-Br-CyMe_4_-BTPhen, 5-(4-hydroxyphenyl)-CyMe_4_-BTPhen, and 5-nitryl-CyMe_4_-BTPhen were synthesized for extraction of actinides and lanthanides. The former three ligands exhibited good performance for selectively extract Am(lll) over Eu(lll) from HNO_3_ solution and their distribution ratio for metal ion was in agreement with previous reports. 5-nitryl-CyMe_4_-BTPhen was first developed by us to suppress the extraction of Eu(lll) into an organic phase for efficient separation from Am(lll). The separation factor of Am(lll) and Eu(lll) was enhanced to 870 (HNO_3_ = 1M, (ligand) = 5 mmol/L). The basicity of four BTPhen ligands was investigated by NMR titration in the media of CD_3_OD. Their protonation constants were determined to be 3.1 ± 0.1, 2.3 ± 0.2, 0.9 ± 0.2, and 0.5 ± 0.1, respectively. The tendency of affinity to H^+^ of these ligands follows the order of L_1_ > L_2_ > L_3_ > L_4_, which is inverse to the strength of the withdrawing electron substituent: nitryl (L_4_) > hydroxyphenyl (L_3_) > bromine (L_2_) > no substitution (L_1_). In addition, the stability constants of four BTPhen ligands with Eu(III) were obtained by UV–vis and fluorescence investigation. The results demonstrate that the affinity of these ligands to lanthanides gradually diminishes with the inductive effect by a substituent, which is in accordance with the solvent extraction results. Even though the novel ligands L_4_ shows the best selective extraction performance of actinides over lanthanides from a highly acidic solution, its solubility in hydrocarbon still needs to be improved. The results of this paper are of important reference in new phenanthroline-derived ligand design for industrial processing of used nuclear fuel. Further effort needs to be made in the tuning solubility of hydrocarbon solvents and available stripping (back-extraction) by dilute acid.

## Figures and Tables

**Table 1:** Protonation constant of N-heterocyclics.

Ligands	Log pKa	Solvent	Methods	Reference
1,10-phenanthroline	4.92	water	-	-
BTP	3.3	76%Methanol	NMR	[33]
Terpy	4.33	Water, I = 0.1 M	UV/Vis	[34]
L_1_	3.1 ± 0.1	Methanol	NMR	[24]
L_1_	3.1 ± 0.1	Methanol	NMR	Present Work
L_2_	2.3 ± 0.2	Methanol	NMR	Present Work
L_3_	0.9 ± 0.2	Methanol	NMR	Present Work
L_4_	0.5 ± 0.1	Methanol	NMR	Present Work

**Table 2:** Conditional stability constants of BTPhen ligands with Eu(lll).

BTPhen Ligands	Logβ_12_	Solvent	Methods	Reference
L_1_	12.6 ± 0.4	Methanol (with 3.3 mol% water)	TRLFS	[38]
	15.6 ± 1.0	Methanol (I = 0 M)	UV/vis	[26]
	14.2 ± 0.5	Methanol (I = 0.01 M)	UV/vis	Present Work
	11.5 ± 0.6	Methanol (I = 0.01 M)	TRLFS	Present Work
L_2_	11.5 ± 0.3	Methanol (I = 0.01 M)	UV/vis	Present Work
	12.1 ± 0.8	Methanol (I = 0.01 M)	TRLFS	Present Work
L_3_	8.3 ± 0.6	Methanol (I = 0.01 M)	UV/vis	Present Work
L_4_	7.1 ± 0.4	Methanol (I = 0.01 M)	UV/vis	Present Work
	8.6 ± 0.2	Methanol (I = 0.01 M)	TRLFS	Present Work

## Sample Availability

Samples of the compounds are not available from the authors upon request.
